# Video-Assisted Thoracoscopic Surgery Versus Tube Thoracostomy with Fibrinolytics for Treatment of Empyema in Children: A Meta-Analysis of Randomized Controlled Studies

**DOI:** 10.3390/children12091225

**Published:** 2025-09-13

**Authors:** Maria Enrica Miscia, Giuseppe Lauriti, Dacia Di Renzo, Valentina Cascini, Gabriele Lisi

**Affiliations:** 1 Department of Medicine and Aging Science, “G. d’Annunzio” University of Chieti-Pescara, 66100 Chieti, Italy; mariaenrica.miscia@asl.pe.it (M.E.M.); gabriele.lisi@unich.it (G.L.); 2 Pediatric Surgery Unit, “Spirito Santo” Hospital of Pescara, 65100 Pescara, Italy; dacia.direnzo@asl.pe.it (D.D.R.); valentina.cascini@asl.pe.it (V.C.)

**Keywords:** empyema, children, video-assisted thoracoscopic surgery, tube thoracostomy, fibrinolytic, randomized controlled studies, systematic review, meta-analysis

## Abstract

**Background**: The British Thoracic Society recommended tube thoracostomy plus intra-pleural fibrinolytics to treat empyema in children in 2005. However, numerous comparative studies have suggested Video-Assisted Thoracoscopic Surgery (VATS) as a first line of treatment for pediatric empyema due to its superior outcomes, including shorter length of hospital stay (LOS). This meta-analysis aimed to compare the following: (1) the LOS for VATS versus fibrinolytics to treat empyema in children; (2) secondary post-operative outcomes (fever, O_2_ support, time taken for chest tube removal, analgesia, complications, failure, and abnormal chest X-ray at follow-up). **Methods**: The study was conducted according to PRISMA guidelines. A systematic search of PubMed, Cochrane, Web of Science, and Scopus was conducted according to PRISMA guidelines. Two independent investigators identified relevant studies, excluding case reports, opinion articles, and gray literature publications. A meta-analysis of randomized controlled trials (RCTs) was performed using RevMan 5.4, with data expressed as mean ± standard deviation (SD). **Results**: Of 1374 abstracts screened, 104 full-text articles were analyzed, and 6 RCTs (345 patients) were included in the meta-analysis. Patients undergoing VATS had significantly shorter LOS compared to those receiving fibrinolytics (9.1 ± 1.8 vs. 11.5 ± 2.5 days, *p* = 0.05). VATS patients also experienced shorter postoperative fever duration (4.2 ± 0.8 vs. 6.9 ± 4.6 days, *p* = 0.007) and earlier chest tube removal (5.0 ± 2.6 vs. 9.5 ± 3.3 days, *p* = 0.01). No significant differences were found between the two groups for other secondary outcomes. **Conclusions**: Children with empyema appear to benefit from VATS compared to tube thoracostomy plus fibrinolytics, with improved outcomes. Further RCTs are needed to corroborate these results.

## 1. Introduction

Empyema is defined as purulent fluid accumulation in the pleural cavity, a frequent complication of community-acquired pneumonias (CAPs) with increasing incidence worldwide [1,2,3,4,5,6,7].

In 1962, the American Thoracic Society proposed the classification of empyema involving three stages of disease progression, which have historically guided treatment timing:

- Stage 1: The exudative phase, characterized by clear fluid without bacteria, rare cells, normal glucose concentration, and normal pH, lasting 24–72 hours from symptom onset.
- Stage 2: The fibrinopurulent phase, lasting up to 10 days, characterized by purulent effusion with high cellularity, bacterial presence, low glucose concentration, pH < 7.2, and loculations.
- Stage 3: The organizing phase, lasting 2–4 weeks, during which fibroblast proliferation forms a peel and causes thickening of the pleura, potentially limiting lung expansion [3,5,6,7,8,9,10,11].

Treatment options have included antibiotic administration alone, thoracentesis, chest drain insertion with or without fibrinolytics, video-assisted thoracoscopic surgery (VATS), or open thoracotomy with pleural decortication, aiming to sterilize the pleural cavity and break loculations, allowing complete lung re-expansion [4,5,10,11,12,13].

Several retrospective studies have compared clinical outcomes after different treatments, and in recent years, fibrinolytics and VATS have been advocated as first-line therapies. However, there is no clear consensus on the management of pediatric empyema [2,4,6,11,12,13,14,15].

Therefore, the aims of our study were to assess the following:

1. The length of post-operative hospital stay (LOS) comparing VATS versus fibrinolytics;
2. Secondary post-operative outcomes (persistence of fever, O_2_ support requirement, need for analgesia, duration of chest tube, failure rate, complications rate, and abnormal chest X-ray findings at follow-up) after VATS versus fibrinolytics therapy.

## 2. Material and Methods

A systematic review of four databases (PubMed, Web of Science, Scopus, and Cochrane) was conducted by two independent investigators using a distinct search strategy. The same authors identified relevant papers on the management of empyema in pediatric patients and excluded case reports, case series with <10 patients, opinion articles, and grey literature studies. The manuscript was prepared according to PRISMA guidelines [16]. A meta-analysis of randomized controlled studies (RCTs) was performed using RevMan 5.3, with results expressed as mean ± standard deviation (SD).

### 2.1. Data Sources and Study Selection

The study was registered on the international prospective register of systematic reviews PROSPERO (registration CRD420251063859) and prepared in accordance with the Preferred Reporting Items for Systematic Reviews and Meta-Analyses (PRISMA) statement (Table 1) [16,17].

Two authors (M.E.M., G.La.) independently searched four databases (PubMed, Web of Science, Scopus, and Cochrane) for studies focusing on the treatment of empyema in children, with a last search date of May 29, 2025. MeSH headings and keywords used were “pleural empyema treatment” AND “children” (Supplementary File S1). The reference lists of eligible studies were also screened for potential cross-references. Case reports, case series with <10 children, opinion papers, experimental studies, and grey literature (theses, reports, conference proceedings, commercial documentations, bibliographies, and non-commercially published official documents) were excluded. Only RCTs were included, according to a stated PICO strategy (Supplementary File S2) [18]. Full-text studies were selected and assessed for relevance by the same two authors, with disagreements resolved by a third author (G.Li.).

### 2.2. Statistical Analysis

Categorical variables were compared using two-tailed Fisher’s exact test or Pearson’s chi-square test. Mean ± SD was calculated for median and range values [19]. Meta-analysis was performed using RevMan 5.4 [20] with a random effects model. The risk ratio (RR) was calculated for categorical variables, and mean differences (MDs) were estimated for continuous variables. Results are reported with 95% confidence intervals (CIs). Data are expressed as mean ± SD. I^2^ values were used to evaluate homogeneity and quantify the dispersion of effect sizes. Funnel plots assessed potential biases. *p* < 0.05 was considered significant.

### 2.3. Quality Assessment

Two authors (D.D.R. and V.C.) independently assessed the risk of bias for RCTs using the Risk of Bias RoB 2 tool [21]. Disagreements were resolved through discussion and agreement with a third author (G.La.). The quality of evidence was graded using the Grading of Recommendations Assessment, Development and Evaluation (GRADE) methodology [22]. Observational studies were evaluated with low quality of evidence. The quality of evidence was decreased in cases of risk of bias, inconsistency, indirectness imprecision, and publication issues. Heterogeneity was evaluated using I^2^ values, with 0–40%, 30–60%, 50–90%, and 75–100% indicating low, moderate, substantial, and considerable heterogeneity, respectively. Optimal information size (OIS), was used to evaluate imprecision, based on 25% relative risk reduction, α error of 0.05, and β error of 0.20 [23].

## 3. Results

**Figure 1:**
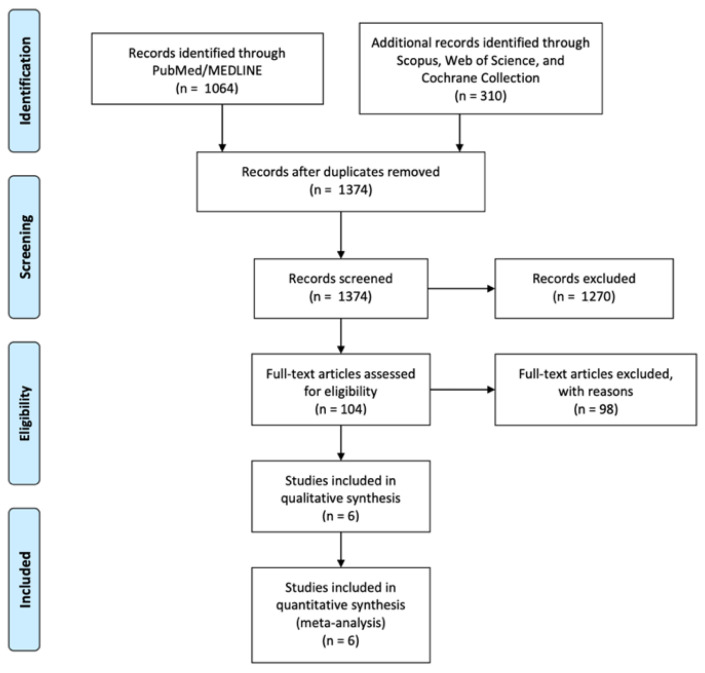
Diagram of workflow in the systematic review and meta-analysis [16].

Of 1374 abstracts screened, 104 full texts were analyzed, and 6 RCTs were included in the meta-analysis (345 patients, Figure 1).

Ninety-eight papers were excluded because they did not meet inclusion criteria (they were not RCTs, did not compare VATS and fibrinolytics, or were not focused on our primary and secondary outcomes).

### 3.1. Systematic Review

A total of 345 patients were included in the study: 173 underwent VATS and 172 underwent fibrinolytics. Urokinase was used as the fibrinolytic agent in 101/172 patients (58.7%) pts, streptokinase in 27/172 cases (15.7%), and tissue plasminogen-activating peptide (tPA) in 44/172 children (25.6%).

The treatment failed in 22/173 cases (12.7%) in the VATS group and in 21/172 patients (12.2%) in the fibrinolytics therapy group (*p* = ns).

Complications were reported in 11/121 cases (9.1%) in the VATS group and in 14/119 patients (11.8%) in the fibrinolytics group. The most reported complication was a persistent pneumothorax in eight patients (three cases in the VATS group and five patients in the fibrinolytics group), followed by subcutaneous emphysema in three cases in the VATS group (Table 2).

### 3.2. Meta-Analysis

**Figure 2:**
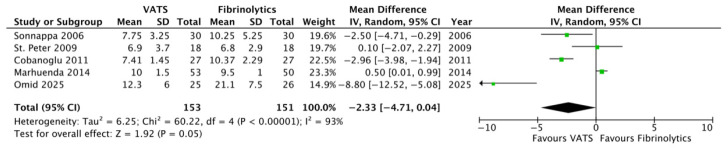
Forest plot comparing the length of hospitalization in the VATS versus the fibrinolytics group [4,6,8,9,24].

Looking at the primary outcome, the length of hospitalization was reduced in patients undergoing VATS compared to fibrinolytics (9.1 ± 1.8 versus 11.5 ± 2.5 days, respectively; *p* = 0.05, MD −2.33, 95%CI [−4.71, 0.04], I^2^ = 93%, Figure 2).

**Figure 3:**
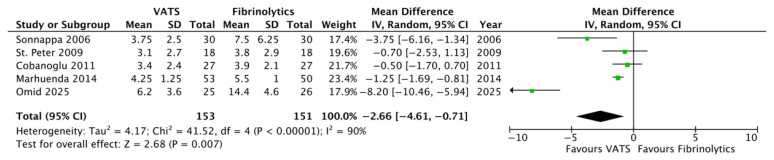
Forest plot comparing postoperative fever in the VATS versus the fibrinolytics group [4,6,8,9,24].

**Figure 4:**
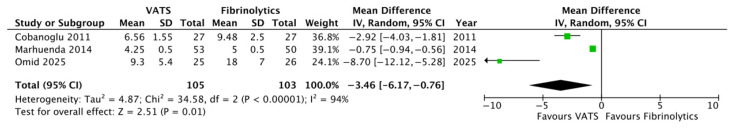
Forest plot comparing the time of chest tube removal in the VATS versus the fibrinolytics group [6,8,9].

**Figure 5:**
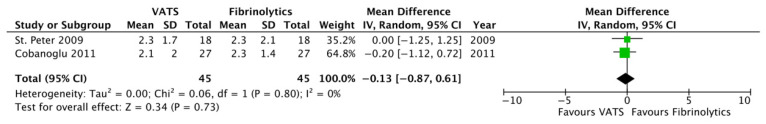
Forest plot comparing O_2_ support in the VATS versus the fibrinolytics group [8,24].

**Figure 6:**
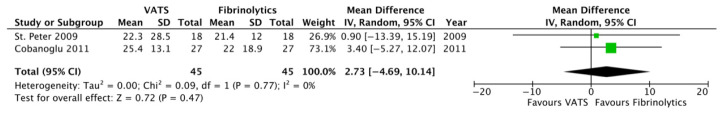
Forest plot comparing postoperative analgesia requirement in the VATS versus the fibrinolytics group [8,24].

**Figure 7:**
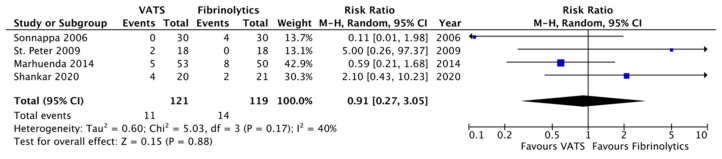
Forest plot comparing post-procedural complications in the VATS versus the fibrinolytics group [4,5,9,24].

**Figure 8:**
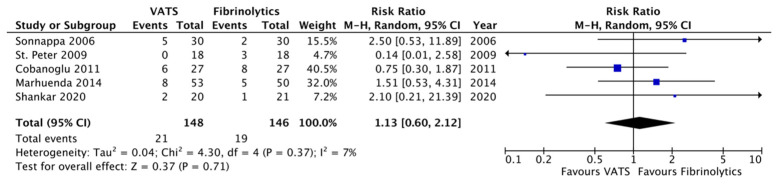
Forest plot comparing failure of therapy in the VATS versus the fibrinolytics group [4,5,8,9,24].

**Figure 9:**
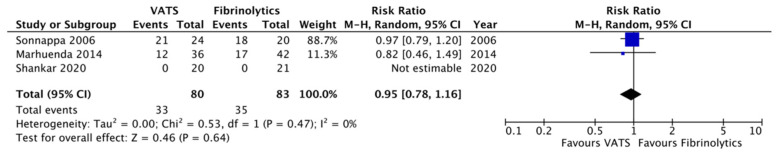
Forest plot comparing abnormal chest X-ray at follow-up in the VATS versus the fibrinolytics group [4,5,9].

Concerning secondary outcomes, the length of post-operative fever was significantly reduced in the VATS group compared to the fibrinolytics group (4.2 ± 0.8 versus 6.9 ± 4.6 days, respectively; *p* = 0.007, MD −2.66, 95%CI [−4.61, −0.71], I^2^ = 90%, Figure 3). Moreover, chest tubes were removed earlier in patients undergoing VATS compared to children undergoing fibrinolytics treatment (5.0 ± 2.6 versus 9.5 ± 3.3 days, respectively; *p* = 0.01, MD −3.46, 95%CI [−6.17, −0.76], I^2^ = 94%, Figure 4). No differences were reported between the two groups with regard to O_2_ support, analgesia, the overall number of post-procedural complications, failure of therapy, and abnormal chest X-ray at follow-up (Figure 5, Figure 6, Figure 7, Figure 8 and Figure 9, Table 3).

## 4. Discussion

The incidence of empyema in the pediatric population is increasing, with up to 50% of community-acquired pneumonias progressing to pleural effusion and empyema [1,6,7,25].

A 2012 study from the APSA Outcome and Clinical Committee reported an incidence rate of para-pneumonic empyema of 7:100,000 in patients under 2 years old, increasing to 10:100,000 in children between 2 and 4 years old [1].

The most common pathogens isolated in Western countries are Streptococcus pneumoniae and Sthaphylococcus aureus [3,5,13]. Despite early diagnosis and improved antibiotic therapy, treatment often proves ineffective, especially in stages 2 and 3 of empyema [8].

Although immunization is the preferred preventive measure, the optimal treatment for parapneumonic effusion (PPE) and empyema remains debated [10].

The primary goal of treatment is to control infection, sterilize the pleural cavity, reduce fever, and allow lung re-expansion [3,4].

Over the past two decades, fibrinolytics treatment and VATS have been advocated as preferred strategies [24].

The British Thoracic Society (BTS) guidelines recommend chest X-ray and thoracic ultrasound as first-line imaging for diagnosis, with CT reserved for complicated cases [1,15].

Treatment options include observation with antibiotic, thoracotomy, chest drain with or without fibrinolytics, and thoracoscopy with or without pleural decortication [8,10,15].

Both VATS and fibrinolytics have shown better outcomes compared to chest tube alone and thoracotomy, but there is no clear evidence of superiority between VATS and fibrinolytics [15].

Proponents of VATS argued that it is more effective in destroying loculations associated with later stages of empyema, resulting in less discomfort and shorter hospital stay [6,24].

In contrast, supporters of fibrinolytics treatment suggest it is equally effective, less invasive, and less expensive than thoracoscopy [4,6,9,24].

Although the BTS recommends tube thoracostomy with fibrinolytics as first-line treatment, reserving VATS for cases of failure, numerous studies have shown comparable long-term outcomes between the two procedures [2,4,5,6,7,8,9,10,13,15,24,25,26].

A recent meta-analysis comparing VATS, fibrinolytics, and chest drain alone found that VATS and fibrinolytics were associated with shorter hospitalizations than chest drain alone. Moreover, fibrinolytics were less expensive than VATS [2]. However, this analysis did not specifically compare outcomes between VATS versus fibrinolytics.

Two meta-analyses of RCTs published in 2010 compared VATS and fibrinolytics.

Krenke et al. found better outcomes with fibrinolytics compared to normal saline. However, no significant difference between were reported comparing fibrinolytics and VATS [27]. Mahant et al. found no difference in LOS between VATS and fibrinolytics, although one study showed reduced hospitalization with VATS [28].

A recent double-blinded RCT, published by Omid et al., found that VATS was associated with shorter hospital stays compared to fibrinolytics, possibly caused by a shorter chest tube duration, antibiotics administration, fever, and dyspnea [6].

Our study, including up to six recent RCTs, had higher statistical power than these previous analyses and found that VATS was associated with shorter hospital stays, quicker fever resolution, and earlier chest drain removal compared to fibrinolytics. However, complications and failure rates were comparable between the procedures, indicating equal effectiveness in resolving empyema.

Our results suggest that VATS might allow for quicker recovery, with equally effective outcomes compared to fibrinolytics.

Even though we did not specifically look at the costs of hospitalization, several studies have shown that VATS might be more expensive and more invasive than fibrinolytic treatment alone; therefore, we can come to the conclusion that fibrinolytic treatment might be preferred from an economic perspective [4,5,9].

### Limitations of the Study

**Figure 10:**
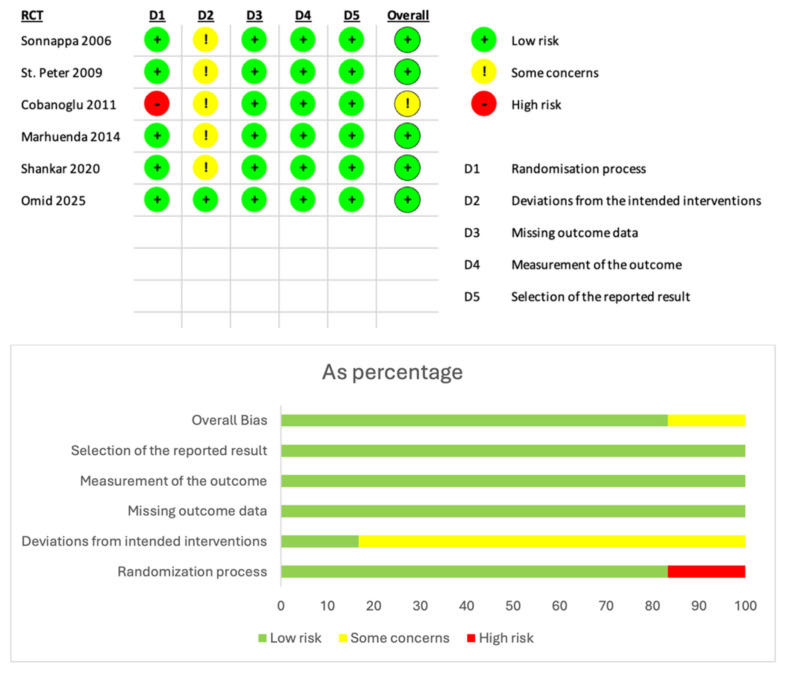
Risk of bias assessment for individual studies [4,5,6,8,9,24] using RoB 2 tool for RCTs [21].

Some limitations were detected, arising from the quality of RCTs included. Power calculation and randomization methodology were not specified in all papers. Only one RCT was double-blinded [6], with possible bias in assessing outcomes. Risk of bias assessment using the RoB2 tool raised concerns in some topics (Figure 10).

The quality of evidence was moderate for LOS, duration of post-operative fever, and failure of the procedure, and low for time of chest tube removal, overall incidence of post-operative complications, and rate of abnormal chest X-ray at follow-up (Table 4). Both the reduced number of patients and significant heterogeneity of data could produce possible bias.

However, two different authors (D.D.R. and V.C.) independently evaluated the present study, thanks to A Measurement Tool to Assess Systematic Reviews (AMSTAR) [29], and the manuscript achieved a trustworthy score (Supplementary File S3).

Finally, the PRISMA checklist was accomplished (Supplementary File S4).

## 5. Conclusions

To our knowledge, this is the first meta-analysis to exclusively include randomized controlled trials comparing fibrinolytics treatment and VATS outcomes.

The results of our study carry a grade A of recommendation.

We found that both procedures have comparable complication and success rates.

However, VATS appears to facilitate quicker recovery by reducing fever duration and chest drain placement time, resulting in shorter hospital stays (level of evidence A).

Further large-scale RCTs are warranted to corroborate these findings.

## Figures and Tables

**Table 1:** Inclusion criteria of the systematic review.

**Publication**	
**Language**	English
**Time period**	January 1984–May 2025
**Subject**	Human studies
**Study type**	Retrospective Prospective Case–control Cohort
**Excluded**	Case reports Case series (<10 patients) Editorials Letters Grey literature
**Keywords**	Empyema Children Video-assisted thoracoscopic surgery tube thoracostomy Fibrinolytic

**Table 2:** Different complications after VATS or fibrinolytic treatment.

Type of Complication	Total (n =)	VATS (n =)	Fibrinolytics (n =)
**Prolonged pneumothorax**	8	3	5
**Drain displacement**	4	0	4
**Subcutaneous emphysema**	3	3	0
**Prolonged hospital stay**	3	1	2
**Ventilatory support requirement**	3	2	1
**Intra-operatory bleeding**	1	1	0
**Bronchopulmonary fistula**	1	1	0
**Extravasation**	1	0	1
**Dyspnea and pain**	1	0	1
**Total**	25	11	14

**Table 3:** Summary of outcomes after VATS versus fibrinolytic treatment of empyema.

Post-Operative Outcomes	VATS	Fibrinolytic	*p* Value
**Length hospital stay (days)**	9.1 ± 1.8	11.5 ± 2.5	0.05
**Fever (days)**	4.2 ±0.8	6.9 ± 4.6	0.007
**0_2_ support (days)**	2.2 ± 0.2	2.3 ± 0.2	ns
**Analgesia (doses)**	24.2 ± 10.9	21.8 ± 1.8	ns
**Time to chest tube removal (days)**	5.0 ± 2.6	9.5 ± 3.3	0.01
**Complications (%)**	9.0 ± 8.2	11.8 ± 7	ns
**Failure (%)**	9.0 ± 8.4	13.0 ± 10.0	ns
**Abnormal chest X-ray at follow up (%)**	41.2 ± 44.2	42.2 ± 45.1	ns

**Table 4:** GRADE evidence profile [22] for the present meta-analysis.

Quality Assessment							No. of Patients		Effect		Quality
No. of Studies	Study Design	Risk of Bias	Inconsistency	Indirectness	Imprecision	Other Considerations	Cases	Controls	Relative (95% CI)	Absolute (95% CI)	
**LOS in VATS versus Fibrinolytics**							**VATS**	**Fibrinolytics**			
5	RCTs	Moderate ^a^	Low	Not serious	Serious ^b^	None	153	151	---	MD 2.33 lower (from 4.71 lower to 0.04 higher)	⊗⊗⊗O MODERATE
**Post-op fever in VATS versus Fibrinolytics**							**VATS**	**Fibrinolytics**			
5	RCTs	Moderate ^a^	Low	Not serious	Serious ^b^	None	153	151	---	MD 2.66 lower (from 4.61 to 0.71 lower)	⊗⊗⊗O MODERATE
**Time of chest tube removal in VATS versus Fibrinolytics**							**VATS**	**Fibrinolytics**			
3	RCTs	Moderate ^a^	Moderate	Not serious	Serious ^b^	None	105	103	---	MD 3.46 lower (from 6.17 to 0.76 lower)	⊗⊗OO LOW
**O_2_ support in VATS versus Fibrinolytics**							**VATS**	**Fibrinolytics**			
2	RCTs	Moderate ^a^	Moderate	Not serious	Serious ^b^	None	45	45	---	MD 0.13 lower (from 0.87 lower to 0.61 higher)	⊗OOO VERY LOW
**Post-op analgesia requirement in VATS versus Fibrinolytics**							**VATS**	**Fibrinolytics**			
2	RCTs	Moderate ^a^	Moderate	Not serious	Serious ^b^	None	45	45	---	MD 2.73 higher (from 4.69 lower to 10.14 higher)	⊗OOO VERY LOW
**Post-op complications in VATS versus Fibrinolytics**							**VATS**	**Fibrinolytics**			
4	RCTs	Moderate ^a^	Low	Not serious	Serious ^b^	None	11/121 (9.1%)	14/119 (11.8%)	RR 0.91 (0.27, 3.05)	27 fewer per 1000 (from 219 fewer to 615 more)	⊗⊗OO LOW
**Failure in VATS versus Fibrinolytics**							**VATS**	**Fibrinolytics**			
5	RCTs	Moderate ^a^	Low	Not serious	Serious ^b^	None	21/148 (14.2%)	19/146 (13.0%)	RR 1.13 (0.60, 2.12)	12 more per 1000 (from 37 fewer to 103 more)	⊗⊗⊗O MODERATE
**Abnormal Chest X-ray at follow-up in VATS versus Fibrinolytics**							**VATS**	**Fibrinolytics**			
3	RCTs	Moderate ^a^	Low	Not serious	Serious ^b^	None	33/80 (41.2%)	35/83 (42.2%)	RR 0.95 (0.78, 1.16)	10 fewer per 1000 (from 44 fewer to 32 more)	⊗⊗OO LOW

**LOS:** length of hospital stay; **VATS:** video-assisted thoracoscopy. ^a^ Bias due to possible confounding; ^b^ OIS not met. GRADE Working Group grades of evidence. **High quality:** Further research is very unlikely to change our confidence in the estimate of effect. **Moderate quality:** Further research is likely to have an important impact on our confidence in the estimate of effect and may change the estimate. **Low quality:** Further research is very likely to have an important impact on our confidence in the estimate of effect and is likely to change the estimate. **Very low quality:** We are very uncertain about the estimate.

## Data Availability

Data supporting this study are available as Supplementary Files.

## Supplementary Materials

The following supporting information can be downloaded at https://www.mdpi.com/article/10.3390/children12091225/s1: Supplementary File S1: Search strategy; Supplementary File S2: PICO strategy; Supplementary File S3: AMSTAR criteria [29] for the present systematic reviews and meta-analysis assessed by two authors; Supplementary File S4: PRISMA checklist.

## References

[1] Islam S., Calkins C.M., Goldin A.B., Chen C., Downard C.D., Huang E.Y., Cassidy L., Saito J., Blakely M.L., Rangel S.J. (2012). The diagnosis and management of empyema in children: A comprehensive review from the APSA Outcomes and Clinical Trials Committee. J. Pediatr. Surg..

[2] Fernandez Elviro C., Longcroft-Harris B., Allin E., Leache L., Woo K., Bone J.N., Pawliuk C., Tarabishi J., Carwana M., Wright M. (2023). Conservative and Surgical Modalities in the Management of Pediatric Parapneumonic Effusion and Empyema: A Living Systematic Review and Network Meta-Analysis. Chest.

[3] Yue F., Yang Z., Yang F., Liu Y., Zhao L., Chen Z., Gao F. (2019). Clinical observation of bronchoscopy alveolar lavage combined with thoracoscopy in the treatment of empyema in children. Medicine.

[4] Sonnappa S., Cohen G., Owens C.M., van Doorn C., Cairns J., Stanojevic S., Elliott M.J., Jaffé A. (2006). Comparison of urokinase and video-assisted thoracoscopic surgery for treatment of childhood empyema. Am. J. Respir. Crit. Care Med..

[5] Shankar G., Sahadev R., Santhanakrishnan R. (2020). Pediatric empyema thoracis management: Should the consensus be different for the developing countries?. J. Pediatr. Surg..

[6] Omid M., Rafieezadeh A., Talebi Anaraki K., Kaviany H., Memarzadeh M., Reisi M., Keivanfar M. (2025). Comparing the efficacy of video assisted thoracoscopic surgery (VATS) vs intrapleural fibrinolytic therapy in children with pleural empyema. Pediatr. Surg. Int..

[7] Mohajerzadeh L., Lotfollahzadeh S., Vosoughi A., Harirforoosh I., Parsay S., Amirifar H., Farahbakhsh N., Atqiaee K. (2019). Thoracotomy versus Video-Assisted Thoracoscopy in Pediatric Empyema. Korean J. Thorac. Cardiovasc. Surg..

[8] Cobanoglu U., Sayir F., Bilici S., Melek M. (2011). Comparison of the methods of fibrinolysis by tube thoracostomy and thoracoscopic decortication in children with stage II and III empyema: A prospective randomized study. Pediatr. Rep..

[9] Marhuenda C., Barceló C., Fuentes I., Guillén G., Cano I., López M., Hernández F., Pérez-Yarza E.G., Matute J.A., García-Casillas M.A. (2014). Urokinase versus VATS for treatment of empyema: A randomized multicenter clinical trial. Pediatrics.

[10] Shatila M., Abu Arab W., Fasih N., Karara K., Ramadan A.M. (2018). Comparative study between outcome of intercostal tube drainage and video assisted thoracoscopic surgery in management of complicated parapneumonic effusion in children. J. Egypt. Soc. Cardio-Thorac. Surg..

[11] Kurt B.A., Winterhalter K.M., Connors R.H., Betz B.W., Winters J.W. (2006). Therapy of parapneumonic effusions in children: Video-assisted thoracoscopic surgery versus conventional thoracostomy drainage. Pediatrics.

[12] Thomson A.H., Hull J., Kumar M.R., Wallis C., Balfour Lynn I.M. (2002). Randomised trial of intrapleural urokinase in the treatment of childhood empyema. Thorax.

[13] Karaman I., Erdoğan D., Karaman A., Cakmak O. (2004). Comparison of closed-tube thoracostomy and open thoracotomy procedures in the management of thoracic empyema in childhood. Eur. J. Pediatr. Surg..

[14] Wang J.N., Yao C.T., Yeh C.N., Liu C.C., Wu M.H., Chuang H.Y., Wu J.M. (2006). Once-daily vs. twice-daily intrapleural urokinase treatment of complicated parapneumonic effusion in paediatric patients: A randomised, prospective study. Int. J. Clin. Pract..

[15] Balfour-Lynn I.M., Abrahamson E., Cohen G., Hartley J., King S., Parikh D., Spencer D., Thomson A.H., Urquhart D., Paediatric Pleural Diseases Subcommittee of the BTS Standards of Care Committee (2005). BTS guidelines for the management of pleural infection in children. Thorax.

[16] Moher D., Liberati A., Tetzlaff J., Altman D.G., PRISMA Group (2009). Preferred reporting items for systematic reviews and meta-analyses: The PRISMA Statement. Open Med..

[17] PROSPERO International Prospective Register of Systematic Reviews. https://www.crd.york.ac.uk/prospero.

[18] Baker A., Young K., Potter J., Madan I. (2010). A review of grading systems for evidence-based guidelines produced by medical specialties. Clin. Med..

[19] Hozo S.P., Djulbegovic B., Hozo I. (2005). Estimating the mean and variance from the median, range, and the size of a sample. BMC Med. Res. Methodol..

[20] Review Manager (RevMan) (2014). The Nordic Cochrane Centre.

[21] Sterne J.A.C., Savović J., Page M.J., Elbers R.G., Blencowe N.S., Boutron I., Cates C.J., Cheng H.Y., Corbett M.S., Eldridge S.M. (2019). RoB 2: A revised tool for assessing risk of bias in randomised trials. BMJ.

[22] Guyatt G.H., Oxman A.D., Vist G.E., Kunz R., Falck-Ytter Y., Alonso-Coello P., Schünemann H.J., GRADE Working Group (2008). GRADE: An emerging consensus on rating quality of evidence and strength of recommendations. BMJ.

[23] Dupont W.D., Plummer W.D. (1990). Power and sample size calculations. A review and computer program. Control. Clin. Trials.

[24] St Peter S.D., Tsao K., Spilde T.L., Keckler S.J., Harrison C., Jackson M.A., Sharp S.W., Andrews W.S., Rivard D.C., Morello F.P. (2009). Thoracoscopic decortication vs tube thoracostomy with fibrinolysis for empyema in children: A prospective, randomized trial. J. Pediatr. Surg..

[25] Livingston M.H., Mahant S., Connolly B., MacLusky I., Laberge S., Giglia L., Yang C., Roberts A., Shawyer A., Brindle M. (2020). Effectiveness of Intrapleural Tissue Plasminogen Activator and Dornase Alfa vs Tissue Plasminogen Activator Alone in Children with Pleural Empyema: A Randomized Clinical Trial. JAMA Pediatr..

[26] Hanson S.J., Havens P.L., Simpson P.M., Nugent M.L., Wells R.G. (2015). Intrapleural alteplase decreases parapneumonic effusion volume in children more than saline irrigation. Pediatr. Pulmonol..

[27] Krenke K., Peradzyńska J., Lange J., Ruszczyński M., Kulus M., Szajewska H. (2010). Local treatment of empyema in children: A systematic review of randomized controlled trials. Acta Paediatr..

[28] Mahant S., Cohen E., Weinstein M., Wadhwa A. (2010). Video-assisted thorascopic surgery vs. chest drain with fibrinolytics for the treatment of pleural empyema in children: A systematic review of randomized controlled trials. Arch. Pediatr. Adolesc. Med..

[29] Shea B.J., Grimshaw J.M., Wells G.A., Boers M., Andersson N., Hamel C., Porter A.C., Tugwell P., Moher D., Bouter L.M. (2007). Development of AMSTAR: A measurement tool to assess the methodological quality of systematic reviews. BMC Med. Res. Methodol..

