# Supplementary material for: Video-Assisted Thoracoscopic Surgery Versus Tube Thoracostomy with Fibrinolytics for Treatment of Empyema in Children: A Meta-Analysis of Randomized Controlled Studies
Source: Children (Basel). 2025 Sep 13;12(9):1225. doi: 10.3390/children12091225 (PMC12468689; doi:10.3390/children12091225)
Supplement: Supplementary file 1 [file children-12-01225-s001.zip › Supplementary file S1.pdf]

**Supplementary file S1:** Search strategy.

**PubMed/MEDLINE**

1. (Video-Assisted Thoracoscopic Surgery).mp.
2. (tube thoracostomy*adj2).mp.
3. (fibrinolytics*adj2).mp.
4. (empyema*adj2).mp.
5. 1 AND 4
6. 2 AND 3 AND 4

**Scopus**

TITLE-ABS-KEY ( ( Video-Assisted Thoracoscopic Surgery ) AND ( tube thoracostomy ) AND ( fibrinolytics ) AND ( empyema ) )

**Cochrane Collaboration**

1. MeSH descriptor : Video-Assisted Thoracoscopic Surgery, tube thoracostomy, fibrinolytics
2. MeSH descriptor : empyema
3. Explode all trees

**Web of Science**

TOPIC ( ( Video-Assisted Thoracoscopic Surgery ) AND ( tube thoracostomy ) AND ( fibrinolytics ) AND ( empyema ) )
