# Supplementary material for: Video-Assisted Thoracoscopic Surgery Versus Tube Thoracostomy with Fibrinolytics for Treatment of Empyema in Children: A Meta-Analysis of Randomized Controlled Studies
Source: Children (Basel). 2025 Sep 13;12(9):1225. doi: 10.3390/children12091225 (PMC12468689; doi:10.3390/children12091225)
Supplement: Supplementary file 1 [file children-12-01225-s001.zip › Supplementary file S2.pdf]

Supplementary file S2: PICO strategy.

**Population**	Children with empyema (age <18 years)
**Intervention**	VATS
**Comparison**	Fibrinolytic therapy (any)
**Outcomes**	PRIMARY: hospitalization<br>SECONDARY: persistence of fever, O_2 support requirement, need for analgesia, duration of chest tube, failure rate, complications rate, abnormal chest X-ray findings at follow-up

	**INCLUSION CRITERIA**	**EXCLUSION CRITERIA**
**Study design**	Randomized Control Trials	Reviews<br>Case series<br>Case/control studies<br>Prospective studies
**Population**	Children (age <18 years) with diagnosis of empyema	Any other pleural effusion
**Intervention**	VATS	Antibiotic therapy alone<br>Thoracotomy
**Comparison**	Fibrinolytic therapy	Tube thoracostomy alone<br>Antibiotic therapy alone
