# Supplementary material for: Video-Assisted Thoracoscopic Surgery Versus Tube Thoracostomy with Fibrinolytics for Treatment of Empyema in Children: A Meta-Analysis of Randomized Controlled Studies
Source: Children (Basel). 2025 Sep 13;12(9):1225. doi: 10.3390/children12091225 (PMC12468689; doi:10.3390/children12091225)
Supplement: Supplementary file 1 [file children-12-01225-s001.zip › Supplementary file S3.pdf]

Supplementary file S3: AMSTAR criteria [29] for the present systematic reviews and meta-analysis assessed by two authors**.**

Item	DDR	VC
1. Was an 'a priori' design provided?	1	1
2. Was there duplicate study selection and data extraction?	1	1
3. Was a comprehensive literature search performed?	1	1
4. Was the status of publication (i.e. grey literature) used as an inclusion criterion?	1	1
5. Was a list of studies (included and excluded) provided?	0	0
6. Were the characteristics of the included studies provided?	1	1
7. Was the scientific quality of the included studies assessed and documented?	1	1
8. Was the quality of the included studies used appropriately in formulating conclusions?	1	1
9. Were the methods used to combine the findings of studies appropriate?	1	1
10. Was the likelihood of publication bias assessed?	1	1
11. Was the conflict of interest included?	0	0
Total	9/11	9/11

0 = No, 1 = Yes
